# Low-Variance Surface-Enhanced Raman Spectroscopy Using Confined Gold Nanoparticles over Silicon Nanocones

**DOI:** 10.1021/acsanm.3c01249

**Published:** 2023-05-20

**Authors:** Dirk Jonker, Ketki Srivastava, Marta Lafuente, Arturo Susarrey-Arce, Ward van der Stam, Albert van den Berg, Mathieu Odijk, Han J.G.E Gardeniers

**Affiliations:** † Mesoscale Chemical Systems, MESA+ Institute, University of Twente, P.O. Box 217, 7500 AE Enschede, The Netherlands; ‡ BIOS, MESA+ Institute, University of Twente, P.O. Box 217, 7500 AE Enschede, The Netherlands; § Inorganic Chemistry and Catalysis, Institute for Sustainable and Circular Chemistry and Debye Institute for Nanomaterial Science, Utrecht University, Universiteitsweg 99, 3584 CG Utrecht, The Netherlands

**Keywords:** gold nanoparticles, silicon nanocones, plasmonics, surface-enhanced Raman spectroscopy, homogeneity

## Abstract

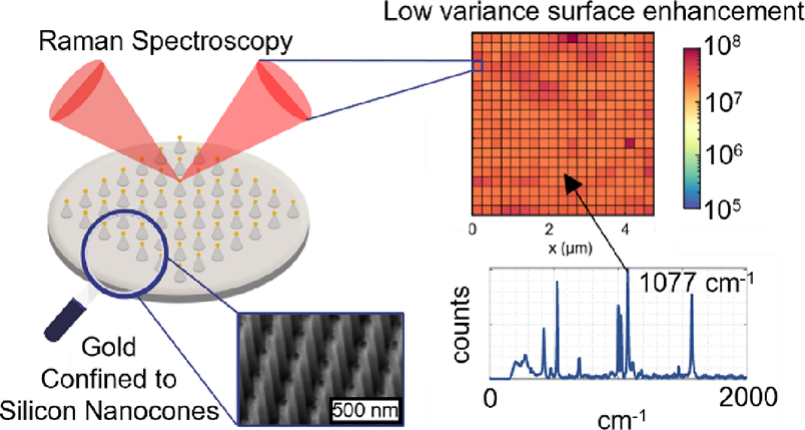

Surface-enhanced Raman spectroscopy (SERS) substrates are of utmost interest in the analyte detection of biological and chemical diagnostics. This is primarily due to the ability of SERS to sensitively measure analytes present in localized hot spots of the SERS nanostructures. In this work, we present the formation of 67 ± 6 nm diameter gold nanoparticles supported by vertically aligned shell-insulated silicon nanocones for ultralow variance SERS. The nanoparticles are obtained through discrete rotation glancing angle deposition of gold in an e-beam evaporating system. The morphology is assessed through focused ion beam tomography, energy-dispersive X-ray spectroscopy, and scanning electron microscopy. The optical properties are discussed and evaluated through reflectance measurements and finite-difference time-domain simulations. Lastly, the SERS activity is measured by benzenethiol functionalization and subsequent Raman spectroscopy in the surface scanning mode. We report a homogeneous analytical enhancement factor of 2.2 ± 0.1 × 10^7^ (99% confidence interval for *N* = 400 grid spots) and made a comparison to other lithographically derived assemblies used in SERS. The strikingly low variance (4%) of our substrates facilitates its use for many potential SERS applications.

## 1 Introduction

Surface-enhanced Raman spectroscopy (SERS) has emerged as a well-established and reliable technique for sensing of analytes. SERS is frequently used in the field of biological and chemical diagnostics.^1,2^ Since its discovery in the 1970s, researchers have improved this technique to provide high orders of enhancement, enabling analyte sensing down to the femtomolar regime.^3^ Nowadays, SERS-based sensing serves as an attractive detection tool, which has been successfully applied to early-stage cancer detection,^4^ single-molecule sensing,^5^ and detection of chemical warfare agents,^6^ and has emerged as a relevant technique in fields such as food safety,^7^ in vitro point-of-care diagnostics,^8^ and electrocatalysis.^9,10^

SERS relies on the basic mechanism of enhancing the unique vibrational fingerprint of a molecule when it is located in the vicinity of electromagnetic hotspots created by metallic nanomachined or nanoroughened surfaces.^11,12^ These hotspots are localized in nanometric volumes in which the enhanced electric field is extremely high. The enhancement mainly depends on (i) physical parameters such as choice of material, 2D or 3D geometry, periodicity of the nanostructures, and their surrounding environment and (ii) the charge transfer between the molecule and the nanostructure due to the overlap of the incident beam wavelength and the absorption plasmon resonance.^11^ A common terminology used to compare the performance of various SERS sensors is the enhancement factor (EF).^13^ While in Finite-Difference Time-Domain (FDTD) simulations, this is expressed as the ratio of enhanced electric field and incoming electric field (|*E*|/|*E*_0_|)^4^ in experimental setups, however, the EF is expressed as the ratio of enhanced SERS spectra of a molecule on a SERS nanostructure to its neat Raman spectra (i.e., without the SERS nanostructure).^14^ Typical SERS EFs are between 10^4^ and 10^7^,^15−17^ but they can go as high as 10^11^.^18^ Having a high EF ensures an increased probability of detecting the analyte of interest. It also enables sensing of analytes at low concentrations (picomolar, femtomolar or lower), thereby lowering the limit of detection.

The field of SERS substrates is rapidly advancing, with new developments continuously emerging. Recent advancements include the development of substrates with higher EF and improved fabrication techniques. However, several challenges still need to be addressed, including reproducibility and scalability. It is worth noting that achieving a homogeneous EF across the entire sensing area is just as important as achieving a high EF.^19^ However, developing SERS substrates with a high, reproducible, and homogeneous EF is still a significant challenge. Having a non-homogeneous substrate leads to large variations in the EF, thereby decreasing the average performance of the SERS substrate.^20^ In most cases, the spectra obtained will be dominated by species present in the high EF areas of the substrate and information regarding other species of interest located in lower EF areas of the substrate will be lost.

Approaches for producing SERS substrates largely rely on lithographic techniques that can be employed for the fabrication of highly stable, reproducible, and periodic SERS substrates.^2^ One of the key advantages of lithography is the flexibility in altering parameters such as shape, size, and spacing with precision down to the nanometer scale.^2^ The past decade has seen an immense increase in the number of nanostructures that can be fabricated for SERS. Geometries such as nanostars, nanodiscs, microbowl arrays, nanotips, and nanodiamonds have been possible with the advent of various nano- and micromachining methods.^2^ While techniques such as electron beam lithography, focused ion beam milling, and laser interference lithography are widely used for the fabrication of nanostructures for SERS, a new emerging technology in this field is displacement Talbot lithography (DTL).^21^

DTL uses an ultraviolet beam to pattern highly ordered nanostructures using a phase shift mask.^22^ It relies on the formation of a Talbot pattern when a periodic grating is illuminated using a monochromatic light source.^21^ An additional displacement of the mask eliminates the problem of a small depth of focus. Since the Talbot pattern is projected over the entire wafer surface, DTL offers fast and reliable wafer-scale fabrication of periodic nanostructures such as lines and dots. Combining DTL with other innovative nanomachining processes has led to the fabrication of a variety of nanostructures, especially for SERS. Le-The et al. combined DTL with a shrink etching technique to achieve nanostructures smaller than 100 nm.^23^ Jonker et al. also report the combination of DTL and subtractive hybrid lithography to obtain selective patterning of a wafer.^24^ This selective patterning allows the fabrication of other components such as electrodes, channels, and attenuated total internal reflection elements around the DTL exposed sensor areas.^25^ The inability to perform sectional exposures can be tackled with a few innovative steps in the fabrication process flow as shown in the work of Le-The et al. and Jonker et al.^23,25^ This makes DTL an enabling technique for SERS substrate fabrication.

Glancing angle deposition (GLAD) has been used for the deposition of randomized nanoparticle arrays.^26^ It is also found to be very useful for the fabrication of column and film microstructures by employing the concept of ballistic shadowing.^27^ Consequently, the technique has enabled the fabrication of nanostructures such as tilted nanorods, helical nanorods, and multilayer nanorods.^28^ However, a system capable of not only tilting the substrate during deposition but also allowing substrate rotation is still relatively new and less explored. Nanodiscs^17,29^ and hemispherical gold substrates^30,31^ are quite common in the literature. They can be fabricated via chemical and lithographic routes. It has, until now, been very difficult to form periodic arrays of spherical gold nanoparticles (Au-NPs) via lithographic routes. A spherical geometry has the advantage of providing a high relative surface area, and when arranged in a highly ordered array, such metallic nanoparticles have great potential for catalytic applications.^32^ So far, there have been very few attempts at fabricating Si-Au nanocone–nanoparticle arrays. Some articles, which might have reported highly periodic and ordered nanocones,^33^ lack the Au-NP aspect, and those that have been able to form nanoparticles miss out on an ordered substrate base.^34^ Having both an ordered nanoparticle array and a periodic nanocone geometry is essential to have a homogeneous EF for SERS-based sensing.

In this work, we report the fabrication of nearly Au-NPs onto ordered silicon nanocone (SiNC) arrays fabricated via a novel combination of DTL and GLAD. This leads to the fabrication of a highly homogeneous SERS substrate with an exceptionally low variance of the enhanced SERS signals. Using the suggested method, we can deposit Au at glancing angles as high as 85° in addition to deposition at selected faces of the SiNC structure, utilizing the ballistic self-shadowing effect. By controlling the deposition parameters, the particle diameters of the Au-NP can be varied. To homogenize the Au-NPs at the SiNC apices (Au-NP@SiNC), the Au-NP@SiNC arrays were subjected to thermal annealing. However, it was observed that annealing leads to the dislocation of the Au-NP away from the SiNC apices, which is detrimental to the EF. For the GLAD deposition without thermal annealing, we obtained the most reproducible results and report an analytical EF (AEF) of (2.2 ± 0.1) × 10^7^ (99% confidence interval for *N* = 400) as calculated by the stretching mode of the C–S bond (ν C–S) located at 1077 cm^–1^. A table is presented in the body of this work that evaluates the performance of lithographically derived SERS substrates. When compared to other structures reported in the literature, the strikingly low variance and highly homogeneous AEF is probably due to the homogeneity of the periodic silicon nanocones. This directly relates to the necessity of a periodic array. In conclusion, the results highlight the potential use of this substrate for in situ SERS characterization during molecular sensing with the advantage of having extremely reliable results due to the remarkably high homogeneity of the fabricated SERS substrate.

## 2 Methods

### 2.1 Silicon Nanocone Fabrication

SiNC fabrication was done according to the method described in Jonker et al.^35^ The method relies on an additive hybrid lithography step including DTL. The mask is patterned using reactive ion etching (RIE) in a nitrogen plasma (TEtske, home-built system).^23^ The mask pattern is transferred into a monocrystalline (100) p-type silicon substrate using inductively coupled plasma RIE (Estrelas, Oxford Instruments) with a mixed-mode sulfur hexafluoride and octafluorocyclobutane gas. The resultant silicon nanowires are sharpened using a combination of argon ion beam etching (i300, Oxford Instruments) and dry thermal oxidation (Omega junior, Tempress) to yield SiNC structures organized in a square periodic array with 250 nm pitch. SiNC arrays are patterned at the wafer scale with mm^2^ array dimensions spaced several cm apart.

### 2.2 Electron Beam Evaporation

Au deposition on SiNC is carried out by an electron beam evaporator (BAK 600, Balzers). It operates at a high vacuum with a process pressure of <1 × 10^–6^ mbar. The deposition system uses a high voltage electron beam for resistive heating of the material past the melting temperature, thereby generating a vapor flux. An acceleration potential of 10 kV directs electrons, originating from a heated filament, toward the Au metal target, contained in a tungsten crucible, in a beam with a spot size much smaller than the crucible diameter. For Au deposition, a beam current between 250 and 280 mA was maintained during deposition. This yields a deposition rate of 0.1 nm/s measured by a quartz crystal mass balance simultaneous to deposition. Thickness values mentioned throughout the article are based on the product of the deposition time and deposition rate.

### 2.3 Annealing

A PID-controlled thermal furnace (TSD-12 furnace, Toma) is used for annealing experiments in air. Experimental annealing temperatures were varied between 800 and 1000 °C. The method comprises a 20 °C/min ramp to the operating temperature, annealing at the said temperature for a set given time and passive cooling down. A tube furnace (Nabertherm GmbH) is used for N_2_ annealing, and prior to filling with N_2_, the chamber is evacuated to a pressure <1 × 10^–3^ mbar.

### 2.4 Finite-Difference Time-Domain Simulations

Numerical simulations are performed on Ansys Lumerical 2021 R1.4 Finite Difference IDE. Visualization of the enhancement is obtained by creating a simulation region around the nanocone and Au-NP. The dimensions for the nanocone geometry are taken from TEM measurements.^35^ The SiNC with an Au-NP (Palik model^36^) is placed in an FDTD simulation region with a pitch of 250 nm in the *x*- and *y*-directions. The SiNC structure is connected at the bottom to a bulk c-Si domain with a length and width (*x*, *y*) of 1 μm. The bulk domain size in the *z*-direction (depth) is systematically varied in a convergence study to accommodate the SiNC, Au-NP, light source, and reflectance monitor (4–8 μm). Periodic boundary conditions are applied in the *xz*- and *yz*-plane boundaries, while perfectly matched layer (PML) boundary conditions are used for the *xy*-plane boundaries. PML boundaries are used as they absorb all incident light without creating any back reflection. A total field scattered field source propagating in the *z*-direction with an incident beam wavelength of 633 nm is used to match the experimental laser wavelength. A profile field and power monitor in the *xy*-plane boundary are placed above the source to collect reflection data. Another monitor is placed in the *yz*-plane at the center of the cone in order to visualize the field enhancement at the cross section of the cone. The entire simulation is run at a mesh accuracy of 2 and mesh size of 0.1 nm. Data analysis is performed in MATLAB.

### 2.5 Reflectance Measurements

A Leica DM600 FS microscope with a 50× magnification and 0.9 NA is used to measure the reflectance spectra of the nanocone samples. The microscope is fitted with a Leica Type 11307072060 bright light source and Optics HR 4000 detector. Reflection measurements of SiNC, Au-NP@SiNC, and Au-NP@SiNC annealed in air at 800 °C and Au-NP@SiNC annealed in air at 1000 °C are performed using this setup. A flat Si substrate was used for reference measurements.

### 2.6 Raman Spectroscopy Measurements

Raman measurements are performed on a (WITec Alpha300R) Raman microscope with a UHTS 300 spectrometer. A 633 nm laser is used in combination with a 100× magnification and 0.9 NA to excite the SERS nanocone substrate. The Raman microscope is operating on the backscatter configuration where the reflected beam is filtered using an edge filter before being directed toward a CCD detector cooled at −60 °C. The Raman spectra of all samples are collected at an integration time of 3 s with a laser power of 2 mW unless stated otherwise. A total of 10 measurements are performed, and the final averaged spectrum was used for data analysis. Square mapping is conducted at 20 by 20 square gridpoints (1 grid point = 250 nm × 250 nm) yielding 400 measurements spanning different surface areas.

Benzenethiol (BT) (99%, Thermo Fischer) is used as a probe molecule due to the strong and fast binding of Au-SH bonds forming a conformal monolayer at the Au surface. The Au-NP@SiNC samples (QD, QD_800_, QD_1000_; defined in 3.6) are first immersed in a 10 mM BT-in-ethanol (96%, Thermo Fischer) solution for 12 h, then cleaned with fresh ethanol, and dried under a N_2_ stream. For reference measurements, an undiluted (pure) BT solution is used and contained in a glass capillary. The reference spectra are obtained under the same microscope settings as that of the other samples.

### 2.7 Enhancement Factor Calculation

Prior to AEF calculation later discussed in the Results and Discussion section, the spectra are baseline-corrected, using the asymmetrically reweighted penalized least squares smoothing (arPLS) algorithm,^37^ with lambda = 10^4^ and *p* = 0.001, smoothened with a moving mean filter with window size 3. The Supporting Information of this study includes a discussion on the impact of implementing arPLS baseline correction and moving average filter on the observed variance. Applying these corrections to individual recorded spectra increases the total observed variance because the variance statistic combines the individual variance terms, which include variance introduced by the measurement system and variance introduced by the mathematical treatment of each individual spectrum. The AEF is calculated by considering the peak value of the selected Raman band (1077 cm^–1^) of the SERS substrate, *I*_SERS_, to the peak value recorded in the pure BT solution, *I*_RAMAN_, according to
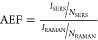
1

The peak values of the SERS and Raman signal were normalized by the number of probed molecules, *N*_SERS_ and *N*_RAMAN_, respectively. For *N*_SERS_, the probed number of molecules depends on the surface density of adhered molecules, σ_BT_, the surface area of the projected incident laser, *A*_Beam_, and the ratio, *G*, of the total surface area of gold present in a unit cell, *A*_Au_, to the area of the unit cell, *A*_unit_. *N*_RAMAN_ depends on the molar concentration of the probed liquid, ρ_BT_, and the volume probed.

In this case, we assumed an average cylindrical volume with a circular surface area equal to the beam area of the laser, *A*_Beam_, and height, *h*, being the interaction length of the laser. Inserting the variables into eq 1 yields
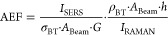
2
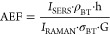
3

With ρ_BT_ = 5.85 × 10^21^ cm^–3^, the product of the molar mass and density of liquid BT, *h* = 1.84 × 10^–4^ cm, σ^BT^ = 3.2 × 10^14^ cm^–2^, *A*_Au_, is the area of the spherical Au-NP resting on top of the SiNC structure with a radius of 34 nm derived from SEM images. *A*_unit_ is the area of the square periodic SiNC structure with pitch 250 nm, giving *G* = 0.06. After filling in the constant, eq 3 evaluates to AEF ≅ 5.8 × 10^4^ × *I*_SERS_/*I*_RAMAN_.

## 3 Results and Discussion

### 3.1 Fabrication of Shell-Isolated Silicon Nanocones

The fabrication of the SiNC array was briefly discussed in the Methods section and based on the work presented by Jonker et al.^35^ The nanocones are organized in a square periodic lattice with 250 nm pitch, base diameter of 100 nm, and cone height of ∼550 nm. The apex of the silicon nanocone has a tip radius of curvature of ∼3 nm and the cone opening angle ∼14°. In this work, we choose to keep the silicon dioxide shell remnant from the dry thermal oxidation sharpening procedure. The thickness of this thermally grown silicon dioxide (t-SiO_2_) is ∼5 nm near the apex of the nanocone and ∼ 20 nm at the base of the nanocone as concluded from transmission electron microscopy (TEM).^35^

### 3.2 Discrete Rotation Glancing Angle Deposition

**Figure 1:**
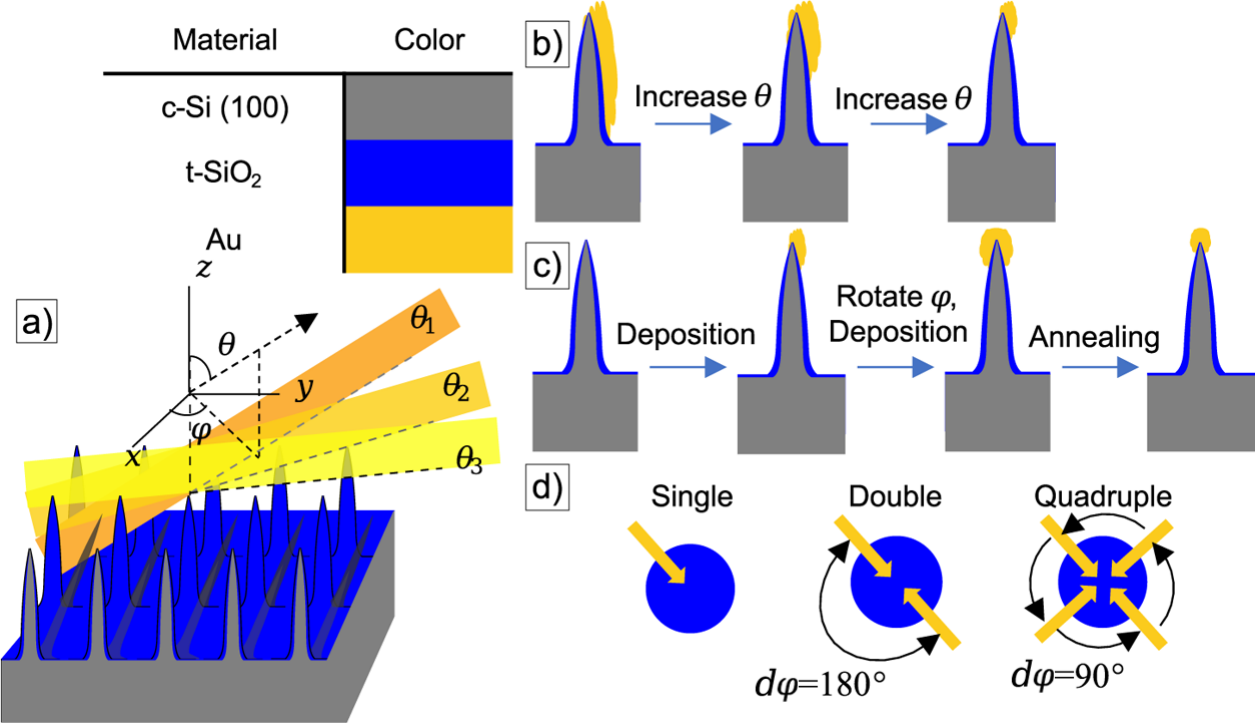
(a) Schematic presentation of the DR-GLAD of Au on SiNCs. The color legend indicates the materials used. Geometric parameters are described relative to the surface normal, *z*. Here, θ is the incidence angle during deposition (also considered as the tilt of the substrate) and φ is the substrate rotational angle. (b) Impact of increase of θ (θ = 60°, 75°, and 85°), confining the deposited Au toward the apex of the SiNC array. (c) Schematic representation of DR-GLAD, which consists of the deposition of Au on the SiNC structure, a dφ = 180° substrate rotation and another deposition of Au, and a final annealing step. (d) Schematic representation of dφ substrate rotation for single, double, and quadruple depositions.

The basic procedure, as presented in Figure 1, involves thermal evaporation of Au under a glancing angle onto an array of SiNC structures consisting of a single crystalline silicon (c-Si) core with a t-SiO_2_ shell. The glancing angle is defined as an oblique incidence angle, θ, between the surface normal of the crucible containing the metal to be evaporated and the surface normal of the substrate onto which the metal is to be deposited, represented as *z* in Figure 1a. The thermal evaporator used in this work has a substrate holder that can be manually rotated prior to deposition for tilt angles 0 ≤ θ ≤ 180° around the *x* axis. Additionally, the substrate can also be rotated manually along the *z* axis prior to the deposition with substrate rotation angles, 0 ≤ φ ≤ 360°. By increasing θ, the deposition of Au is confined toward the apices of the SiNC, schematically represented in Figure 1b. A deposition run can be followed by a thermal annealing step, as shown schematically in Figure 1c. Changing φ allows the selective deposition of the Au layer onto various nanocone faces, as schematically presented in Figure 1d. Here, dφ is a discrete substrate rotation around the *z* axis prior to deposition.

### 3.3 Single Deposition

**Figure 2:**
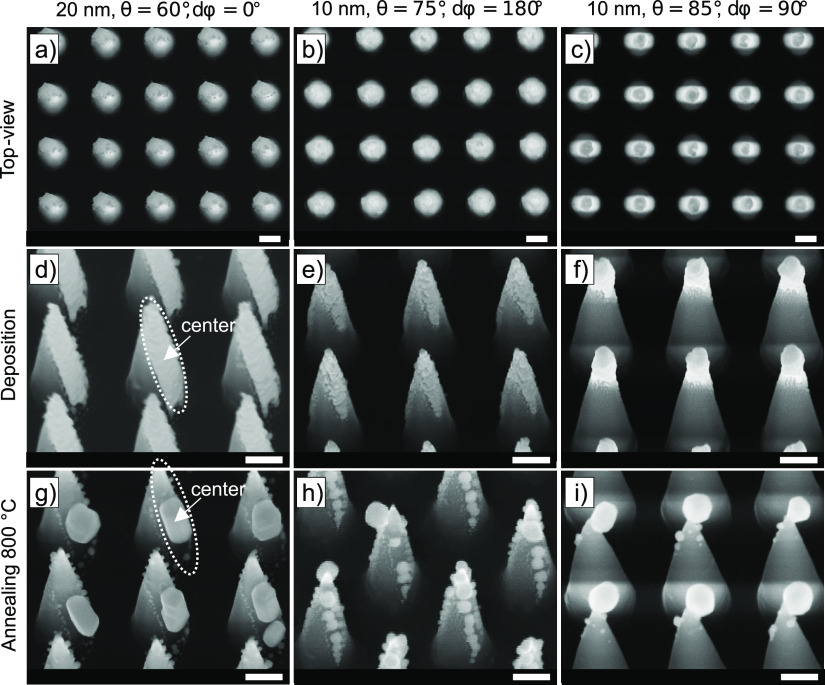
SEM images where the column text title denotes the thickness of the Au deposited, the θ under which the deposition is conducted, and the substrate rotational angle, dφ, for each deposition run. (a–f) SEM images recorded directly after deposition, with top-view (a–c) and 20° tilt angle (d–f) SEM images. The bottom row contains SEM images collected directly after annealing of the samples in air at 800 °C. The scale bars represent 100 nm.

Figure 2 contains the results obtained by scanning electron microscopy (SEM) for experiments conducted through the method described in Figure 1. For the first experiment, a single GLAD of a 20 nm-thick Au layer at θ = 60° is performed. It is observed that a closed film on one face of the SiNC is formed, as shown in Figure 2a,d,g. To form a localized particle from the deposited film, an annealing step in air at 800 °C is conducted for 10 min. Due to surface de-wetting, the metal layer withdraws to a spherical nanoparticle in order to reduce its surface energy. It should be noted that reformation of a Au-NP can be achieved at significantly lower temperatures than the melting temperature of the bulk Au crystal (1063 °C), which is why particle formation is observed even at 800 °C.^38,39^ From the SEM image provided in Figure 2g, it can be confirmed that the nanoparticle formation indeed takes place, although it does so at the center of the deposition-exposed SiNC face. Previous studies in which metal nanoparticles have been formed using annealing also report similar results.^40,41^ Here, the Au atoms start to retract toward the center of the deposition area, leading to the formation of an agglomerated particle.^38^ Two mechanisms contributing to this particle formation are Ostwald ripening and coalescence.^41^ In Ostwald ripening, the particle grows at the expense of atom migration from the smaller particles to the bigger particle, whereas coalescence is the agglomeration of the smaller nanoparticles to reduce the total interfacial energy.^38^ This coincides with the general phenomenon of surface de-wetting where the Au atoms prefer to form bonds to reduce their energy and minimize surface contact area resulting in the formation of one big particle over multiple smaller particles. While it might be difficult to prove which mechanism is the dominating one, it can be assumed that both play a crucial role in forming the Au nanoparticle on the SiNC.

### 3.4 Double Deposition

To influence the deposition more toward the apex of the SiNC, a double deposition discrete rotation (DR-GLAD) is employed by conducting depositions with dφ = 180° between two sequential depositions. Additionally, θ is increased to 75° while the thickness of the deposited film is reduced to 10 nm for individual depositions. The results are shown in Figure 2b,e. After the deposition is performed, the SiNCs are annealed in air at 800 °C for 10 min. The results of this experiment are shown in Figure 2h where the Au-NP formation at the SiNC apices only takes place for some particles while most of the Au-NPs are scattered across the SiNC slanted sidewall. We hypothesize that the deposited Au is too thin to form a closed film. Although the deposition thickness is set to 10 nm, which usually results in the deposition of a closed film over a flat surface, the incident flux on the surface of the nanocone using GLAD is a non-linear function of the θ, which therefore results in less deposited Au than expected.

Figure S1 contains results of additional annealing experiments. All deposition conditions are kept identical. However, the annealing step is now performed in air at 1000 °C for 60 min. This leads to faceted Au-NPs forming at various positions along the SiNC structure. In addition to this, annealing also results in the oxidation of the c-Si core of the SiNC. This can be clearly seen from the expansion of the cone diameter and reduction of the distance between the t-SiO_2_ interfaces of the nearest-neighbor SiNCs. To verify this, the sample is submerged in 50% aqueous HF to remove the t-SiO_2_. The result in Figure S2 shows that the SiNC array is completely etched, indicating that the nanocone consists solely of t-SiO_2_ after annealing for 60 min in air at 1000 °C.

### 3.5 Quadruple Deposition

Figure 2c,f,i contains results of experiments in which a quadruple DR-GLAD is applied and sequential annealing is performed. The quadruple deposition consists of four sequential depositions with three dφ = 90° substrate rotations in between to perform the deposition at four faces of the nanocone. Furthermore, a 10 nm Au film is deposited in each deposition and the sample tilt was increased to θ = 85°. It is found that quadruple deposition is optimal for the formation of Au-NPs at the nanocone apex. Deposition, in this case, is strikingly different when compared to previous cases and yields ordered Au-NPs with a diameter of 67 ± 6 nm confined to the SiNC apices, as extracted from automated Au-NP diameters using MATLAB and shown in Figures S3 and S4.

**Figure 3:**
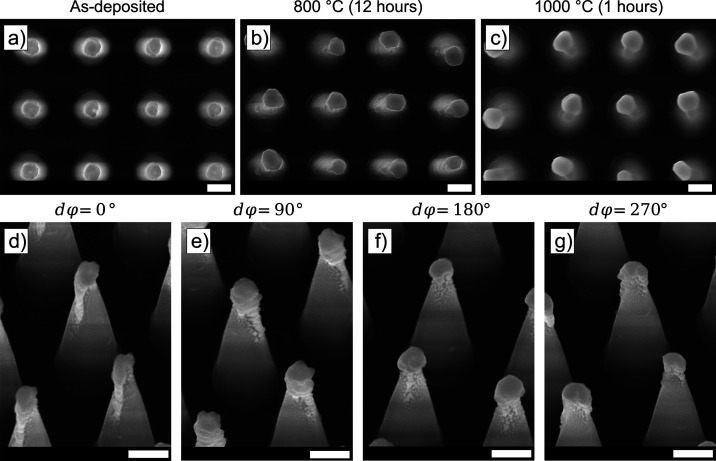
Quadruple deposition: top-view SEM images collected from (a) as-deposited structure with no annealing, (b) as-deposited structure with annealing at 800 °C for 12 h in air, and (c) as-deposited structure with annealing at 1000 °C for 1 h in air. (d–g) SEM images collected for Au-NPs on SiNC (no annealing) fabricated via quadruple deposition of 10 nm Au at θ = 85° through dφ = 90° substrate rotations. SEM images were collected for four discrete substrate rotations, visualizing the DR-GLAD process on different faces of the SiNC structure. The scale bars represent 100 nm.

To homogenize the Au-NP particle size and further confine the particle to the apices, additional annealing experiments are carried out. The first as-deposited sample is annealed in air at 800 °C for 12 h while the second as-deposited sample is annealed at 1000 °C for 1 h. The results are shown in Figure 3 through top-view SEM images. The sample annealed at 1000 °C shows a significantly different morphology compared to the as-deposited and sample annealed at 800 °C. As seen in Figure S5, similar to the double deposition, the Au-NPs become faceted after annealing and tend to reform away from the SiNC apices, an effect which is more pronounced at 1000 °C. Interestingly, another contributing factor could be that the as-deposited Au-NPs display a thin tail as observed under dφ = 0° in Figure 3, possibly moving the deposition center away from the apex. To further characterize this, SEM images were recorded at a 20° tilt angle, for four different substrate rotational angles, each with a 90° difference as shown in Figure 3. Different growth regimes are observed around the periphery of the SiNC structure. We hypothesize that a self-shadowing effect inherent to the DR-GLAD method yields different deposition rates in the φ direction. Herewith, a self-shadowing effect is defined as the situation in which neighboring structures block part of the incident beam. This is possible because the high vacuum conditions during deposition facilitate mean free path lengths several orders of magnitude larger than the distance between nearest neighbor SiNCs. The hypothesis is that the deposition of homogeneous arrays of gold nanoparticles would not be possible if the nanocone array were inhomogeneous, such as in the case of ’black silicon’ or other presented methods. To test this hypothesis, a deposition over the flat surface, also present on the QD sample, is observed using SEM. Figure S7 presents a comparison of SEM images obtained at the flat and SiNC surface. In this case, the flat surface represents the extreme example of the need for a homogeneously structured surface as it is totally absent. Comparing the deposition over the flat surface with that of the SiNC surface, it is evident that the self-shadowing effect in the homogeneous SiNC array is a key parameter for obtaining lower particle size polydispersity. Lastly, because the self-shadow cast by the nearest neighbor is larger than that of the next nearest neighbor, which is on the diagonal of the square periodic unit cell, there is a φ-dependent deposition rate. This effect may cause altered growth regimes when thin layers are deposited. Additional depositions were conducted in which 10 nm Au was in quadruple deposited at θ = 80°, which also present a φ-dependent deposition rate, as shown in Figure S8.

### 3.6 Tomographic Scanning Electron Microscope, Energy-Dispersive X-ray Spectroscopy, and Backscattered Electron Detection

In the following sections, we will focus on the samples shown in Figure 3, for which spatially patterned nanoparticles were obtained closest to the apices of the nanocone structures. The samples are the following: (i) quadruple deposition at θ = 85° with no annealing (QD), (ii) quadruple deposition at θ = 85° with annealing in air at 800 °C for 12 h (QD_800_), and (iii) quadruple deposition at θ = 85° with annealing in air at 1000 °C for 1 h (QD_1000_). The deposition target thickness is 10 nm for each dφ-rotation.

**Figure 4:**
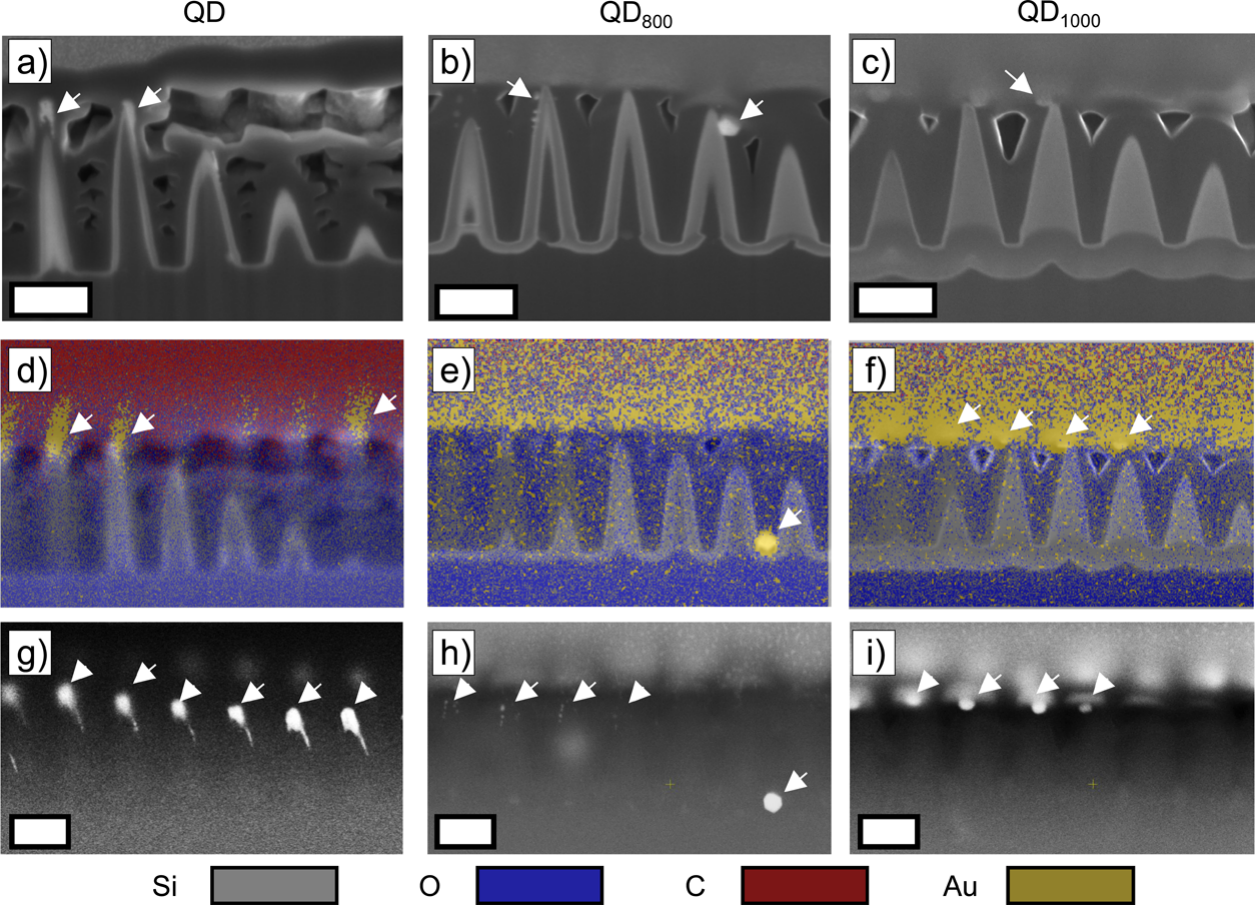
SEM images collected after FIB at the cross section of carbon-coated Au-NPs on SiNC structures after annealing and BT functionalization. (a–c) Collected SEM images at the cross-sections. (d–f) False color images based on the elemental mapping measured through the EDX detector. (g–i) Measured intensity of backscattered electrons. The scale bars represent 250 nm.

To verify the presence of Au-NPs at the apices of the SiNC structure after benzenethiol (BT) functionalization, a combination of focused ion beam (FIB) etching, SEM, and energy-dispersive X-ray spectroscopy (EDX) is used to record images at the cross section of the SiNC array. The SEM data is recorded after functionalization of the Au-NPs in BT and after Raman spectroscopic measurements. However, in this chapter, the tomographic SEM images are discussed prior to the SERS measurements of functionalized Au-NPs. The SERS measurements are discussed in 3.10. The images collected by tomographic SEM are shown in Figure 4.

The most important features observed in the SEM images in Figure 4a–c are that the t-SiO_2_ shell continues to grow during the annealing process in air at elevated temperatures and the precipitation of Au-NPs in QD_800_. The additional oxide growth in annealed samples was verified through secondary effects like the radial expansion of the cone and hydrofluoric acid etching experiments, Figure S2, but is now visualized directly using tomographic FIB + SEM. The continued growth of the t-SiO2 shell can be explained by the presence of water and oxygen molecules in the ambient furnace and the high furnace temperature. At higher temperatures, the oxidation rate increases, which translates to a completely oxidized SiNC structure in the case of annealing at 1000 °C. In this configuration, strictly speaking, there is no SiNC structure anymore because the c-Si core is converted into t-SiO_2_. As can be observed in the EDX maps, Au-NPs are present after all three sample treatments. However, in sample QD_800_, these particles are not positioned at the apices. Considering Figure 3, it can be seen that the Au-NPs were still present prior to BT functionalization. Thus, some interaction during the BT functionalization or specimen preparation may have caused the Au-NPs to detach and precipitate between the SiNC structures, but only in the case of thermal treatment at 800 °C. Using the backscatter detector, as done in Figure 4g–i, localization of a heavy element (observed as a contrast difference in the image) at the apices is observed for QD and QD_1000_. From these measurements, we conclude that Au-NPs are present at the apices of the QD and QD_1000_ samples after functionalization with BT and Raman measurements. It should be noted that the EDX mappings contain both errors from drift during measurements as well as from redeposition during FIB milling; both give rise to excess background Au recorded in the false color images.

Considering Figures 1 to 4, we conclude that an increase in θ confines the incident flux toward the SiNC apex and thus yields confinement of the Au-NP towards the apex of the nanocone. Spheroidal particle formation initiates for θ ≥ 80°. At θ = 85°, a quadruple deposition with target a film thickness of 10 nm results in particle size with an average diameter of 67 ± 6 nm (*n* = 48). In addition to this, the DR-GLAD offers the possibility to tune the Au-NP diameter by varying the deposition thickness. Introducing an automated continuous substrate rotation during deposition might relieve some of the issues regarding the φ-dependent deposition behavior. Moreover, an automated continuous substrate rotation might yield an improved analyses of the deposition characteristics under the various substrate orientations. We show that the unique combination of vertically aligned periodic SiNC structures with the DR-GLAD method facilitates the fabrication of well-defined Au-nps at the SiNC apices forming a homogeneous Si-Au nanocone–nanoparticle array. The results indicate that the DR-GLAD technique only delivers homogeneous nanoparticles if the substrate contains spatially periodic and vertically aligned nanostructures.

### 3.7 Optical Properties of SiNC Supported Au-NP Arrays

To assess the optical properties of the Au-NP@SiNC arrays, reflectance spectrum measurements and FDTD simulations are performed. Measurements of the reflectance spectra are compared to FDTD-simulated reflectance spectra to verify the accuracy of the numerical simulation. Then, the numerical simulation is used to investigate the modal distribution of electromagnetic fields. Finally, the results are related to observations made from Raman spectroscopic measurements.

**Figure 5:**
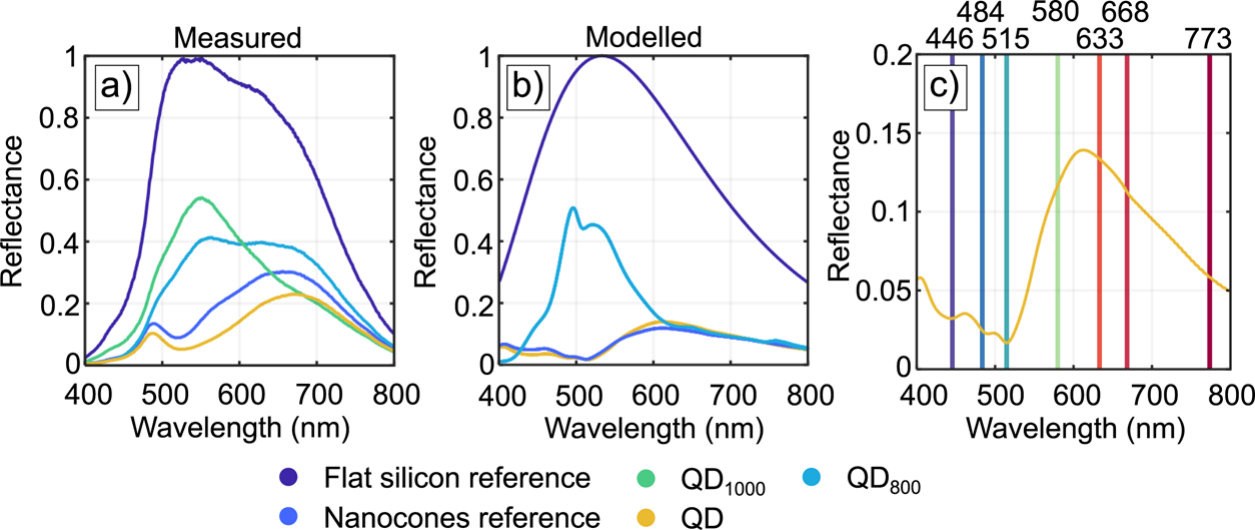
(a) Measured and (b) simulated reflectance spectra for a flat c-Si (100) reference substrate, Au-NP SiNC with no anneal treatment (QD), Au-NP SiNC annealed at 800 °C (QD_800_), Au-NP SiNC annealed at 1000 °C (QD_1000_), and SiNC structures (nanocones reference) without Au-NPs. (c) Magnified representation of the simulated QD sample together with the spectral locations of the minima and shoulders. Electric and magnetic field distributions at these locations are plotted in Figure 6.

Reflectance spectra were recorded directly after annealing and before BT functionalization. Figure 5a shows the measured reflectance spectra averaged over three recorded data points for each wavelength. All datasets were normalized by the largest value recorded on a flat silicon substrate (Si reference). A comparison is made between samples containing only the SiNC structures (nanocones reference), QD, QD_800_, QD_1000_, and the Si reference.

The reflectance spectrum obtained on the Si reference in Figure 5a is a function of the reflectivity of the surface, the responsivity of the detector, and the optical properties of the light source, which is a tungsten halogen incandescent source. Considering the measurement on the Si reference substrate, the recorded reflectance spectrum is predominantly governed by the spectral properties of the light source and no features are observed. Considering the spectra recorded for the nanocones reference and QD sample, a distinct feature is observed with a reflectance minimum around 520 ± 3 nm. To further investigate the optical properties of the SiNC structures and determine the origin of the 520 nm peak, numerical FDTD simulations are performed. During the simulation convergence study, the effect of the thickness of the underlying bulk substrate is also investigated due to the non-linear nature of the silicon absorption coefficient as a function of incident photon energy.^42^ Choosing a too thin substrate thickness results in reflectance from the backside of the silicon substrate and causes a kind of Fabry–Perrot interference, which is evident from the periodic modulation of the reflectance spectra at longer wavelengths, shown in Figure S10. Moreover, this modulation shifts up toward larger wavelengths for larger domains, further underlining the presence of Fabry–Perrot interference modes.^43^ Having established the proper conditions for a simulation domain, four sample treatments, being QD, QD_800_, QD_1000_, and the nanocones reference sample, were simulated of which the simulated reflectance spectra are presented in Figure 5b. When comparing Figure 5a and b, there is a good level of qualitative agreement between the measured and simulated spectra. Certain differences, which may be observed between the measured and simulated spectra, can be expected due to the presence of perfect boundary conditions used in simulations, the experimental polycrystalline nature of the Au forming the Au-NPs, and optical effects during measurement that are not easily captured in a simulated configuration. All these aspects influence differences in the locations and broadness of observed minima in the presented reflectance spectra.

### 3.8 Simulated Magnetic and Electric Field Distributions

**Figure 6:**
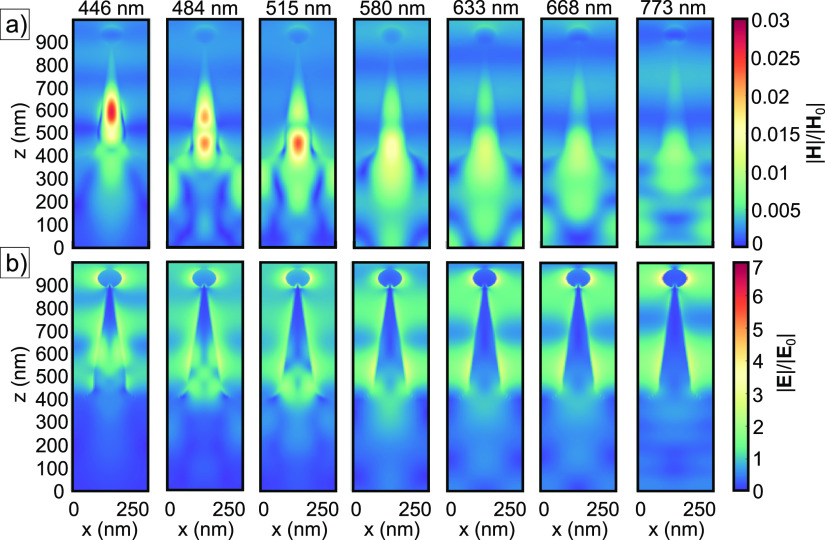
Normalized (a) magnetic and (b) electric field components of the simulated QD sample using a total-field scattered-field light source with different incident beam wavelengths. Cross-sectional views in the *xz*-plane, intersecting the symmetry axis of the SiNC structure, are plotted using a colormap. Only the top-most part of the simulation domain containing the SiNC structure is represented, whereas the 1 × 1 × 20 μm^3^ bulk, sitting below the *z* = 0 plane is not graphically presented.

Typically, if the metallic nanoparticles are within a 2–10 nm neighboring area of each other, the near-field interaction of the surface plasmons gives rise to a near-field enhancement, which can be extremely strong with values reaching as high as 10^11^.^18^ However, for lithographically fabricated structures, the periodicity of the underlying substrate increases the gap between the plasmons to >10 nm. This also gives rise to far-field interactions due to the collective oscillation of the plasmons leading to far-field enhancement. This type of enhancement is typically observed for periodic nanostructures with the key aspect being the homogeneity, which ensures a strong far-field coupling.^44,45^ This indicates that the electric field distribution is an interesting aspect to investigate. Considering the simulated spectra, some minima and maxima shoulders are observed in the QD sample, which are more clear in the zoom-in version of the simulated QD reflectance spectrum in Figure 5c. Distinct minima are observed at 446, 484, and 515 nm, whereas more broadly distributed maxima shoulders are found at 580, 668, and 773 nm. To further understand the optical response of the QD sample, the simulated magnetic and electric field distributions are plotted in Figure 6 at the spectral locations presented in Figure 5c. Additionally, a plot is made for the field distributions at 633 nm, considering the wavelength of the helium–neon laser source used during Raman spectroscopic measurements. In Figure 6, a strong magnetic dipole is observed at 446 nm. For larger wavelengths, e.g., 484 and 515 nm, this magnetic dipole splits into a quadrupole that tends to form two maxima, one toward the Au-NP and one into the substrate. Moreover, a multipolar modal distribution in the electric field component is formed. The distribution shows a resemblance to the anapole mode, theoretically discussed and experimentally observed by Baryshnikova et al.^46^ However, rather than being oriented in the radial and axial directions of the SiNC structures, it is distributed in the vertical, *z*, direction. For increasing wavelengths, this anapole mode seems to be more broadly distributed across the *z*-direction, until finally a maximum of electric field component coincides with the Au-NP. Typical for anapole modes is a minimum of the scattering cross section, σ_sca_, accompanying the modal distribution. This is observed in Figure S9 around 510 nm. Another characteristic of anapoles is the presence of the so-called dark modes. Their presence creates destructive interference in the far-field^47,48^ due to resonance frequency matching of electric and magnetic Mie type multipoles. Usually, this is achieved by tuning the dimensions in symmetric and asymmetric structures.^49^

Different from the anapole state, the observed modal distribution can also be described by the Mie scattering theory. Murphy and Brueck showed that the magnetic dipole resonance wavelength is proportional to the product of the refractive index, *n*, of the particle and its diameter *D* so that λ_MD_ = *nD*.^50^ At 446 nm, the refractive index of crystalline silicon is ∼4.47,^36^ yielding a particle diameter of ∼99 nm, which is close to the diameter of the base of the SiNC structure. For larger incident wavelengths, the refractive index decreases in the case of c-Si, and λ_MD_ increases, accordingly. In the simulated field distribution maps, this would be visualized as a systematic lowering of the magnetic dipole mode into the substrate. However, in Figure 6a, a quadrupole is observed of which the center moves upward toward smaller diameters and decreases in magnitude. This behavior is counterintuitive, when solely considering a Mie scattering model. Nevertheless, the above described observations based on numerical simulations can provide further insight into future design parameters of the nanocones. This might lead to an enhanced far-field interaction of the plasmons culminating in a higher EF, theoretically and experimentally. From the simulations presented in Figure 6b, it can be concluded that in the QD configuration, the theoretical EF is in the order of 10^3^–10^4^ at 633 nm.

### 3.9 Raman Spectroscopy Measurements

To validate the SERS performance of the three QD samples, functionalization with BT is conducted. BT has a relatively large Raman cross section and is therefore regularly used for SERS studies.^51^ In addition to the substrates discussed in the reflectance spectra measurements, the pure BT solution is also measured, and a sputter-deposited Au film functionalized with BT is measured for comparison. The reflectance spectra measurements presented in Figure 5a show that the substrate QD shows a minimum in the reflectance spectrum around 515 nm. From this observation, the most obvious choice for Raman spectroscopy measurements would be to use a laser with a 532 nm wavelength. However, previous experiments showed that gold nanoparticles are prone to heating up significantly at this wavelength, leading to damage. Therefore, instead, the choice was made to use a 633 nm laser.

**Figure 7:**
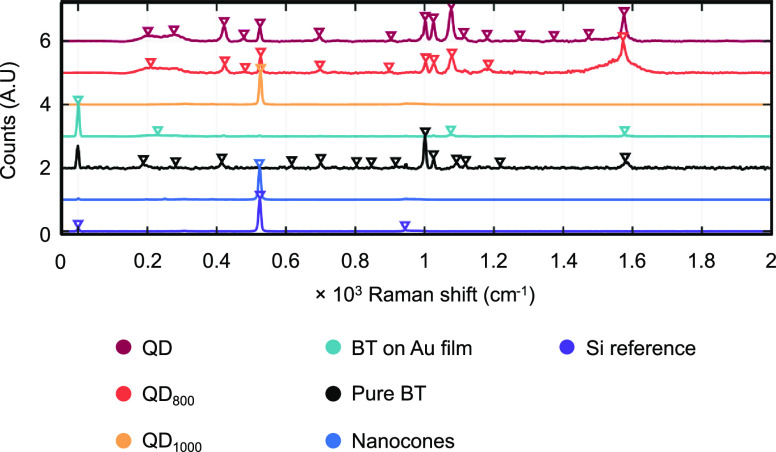
Normalized baseline-corrected and smoothened SERS spectra. Every presented spectrum is the average of 10 recorded spectra. An artificial offset of 1 per dataset was given for visual clarity. The down-pointing triangles indicate the positions of detected peaks using a peak detection algorithm.

**Figure 8:**
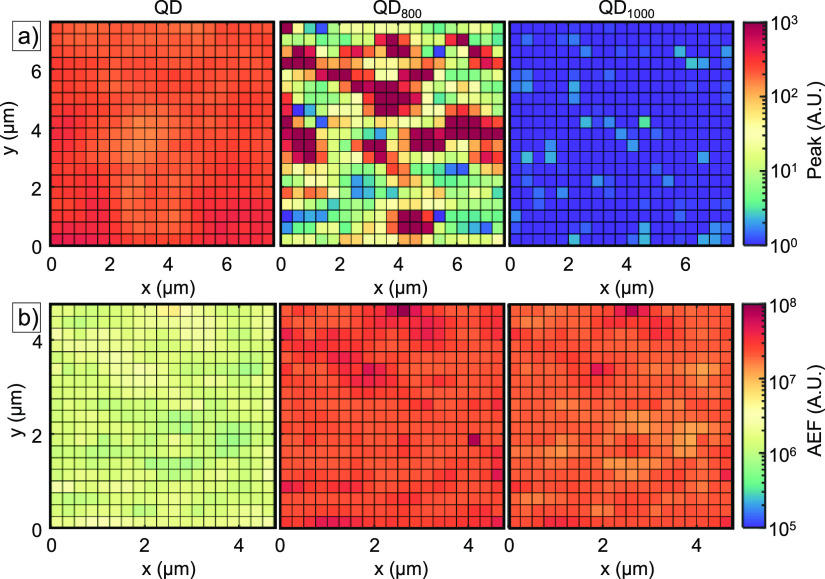
(a) Surface map data for the measured peak values of the baseline-corrected and smoothened SERS spectra at 1077 cm^–1^ for the QD, QD_800_, and QD_1000_ samples plotted as pixels for 400 individual spectra collected over an 8 × 8 μm^2^ surface map with a 20 × 20 grid point measurement. A single measurement was taken for every pixel. (b) Additional surface map data for the QD sample in the form of the calculated AEF for the baseline-corrected and smoothened dataset. Every pixel represents a point calculated at the respective Raman shift, being 1001, 1025, and 1077 cm^–1^, from left to right, respectively.

Figure 7 presents the baseline-corrected and smoothened Raman and SERS signals, normalized at the largest value in the recorded dataset. The smoothened and baseline-corrected spectra for individual samples are presented in Figure S11. The spectra presented for the three Au-NP@SiNC structures QD, QD_800_, and QD_1000_ in Figure 7 were selected from the surface maps plotted in Figure 8a,b based on the highest peak value at 1077 cm^–1^.

The raw and smoothened baseline-corrected datasets of the same sample are presented in Figure S12. Sample QD gives the most homogeneous surface maps, whereas sample QD_800_ shows a large variability with rather large peak values in some spots and low peak values in others. In contrast, no significant peak values are recorded for sample QD_1000_. From the combined FIB-SEM images in Figure 4, it is observed that for sample QD_800_, most of the Au-NPs are moved to the bottom in-between SiNC structures after BT functionalization, whereas a more uniform Au-NP coverage is observed for the QD and QD_1000_ samples. Additional peak value surface maps further evidence this for different Raman shifts in Figure S13.

Whereas enhancement is observed for sample QD, such enhancement is absent for sample QD_1000_. One explanation for the decrease in enhancement for QD_1000_ could be the change in the material of the nanocone from c-Si to t-SiO_2_. When the SiNCs are annealed in air at 1000 °C, complete oxidation of the nanocone leads to a change in the refractive index of the nanocone, thereby quenching the electric and magnetic modes. Much larger particles are needed to support these modes due to a lower refractive index of t-SiO_2_ when compared to c-Si. Moreover, annealing in air at 1000 °C leads to the formation of a tilted and faceted Au-NP at the apex of the nanocone as evidenced by Figure S5. From previous work, it is found that an increasing t-SiO_2_ thickness negatively influences the simulated maximum in normalized electric field magnitude.^52^ We hypothesize that additional tilting of the particle in randomized directions breaks the symmetry of the AuNP@SiNC array, further decreasing the normalized electric field magnitude.

To characterize the SERS performance of the QD sample, first, the analytical enhancement factor is calculated by comparing measurements conducted over the sample surface to measurements in a pure BT solution. Then, a peak detection algorithm is employed to find the Raman vibrational bands observed in the spectra. Figure 8b gives a spatial representation of the calculated AEF for different Raman shifts of BT supported by the QD sample. The Raman shifts are selected both by their corresponding presence in the pure BT solution and the magnitude of the peak value in the baseline-corrected and smoothened SERS spectra. From the surface maps, the mean and standard deviation of the AEF can be calculated, the results of which are shown in Table 1.

**Table 1:** Mean (μ), Standard Deviation (σ), and 99% Confidence Interval (CI) of the Calculated AEF for Different Spectral Locations for Au-NP@SiNC without Annealing Treatment (QD) (*N* = 400) Relative to the Pure BT Solution

Raman shift (cm^–1^)	1001	1025	1077	1576
μ_AEF_ (10^6^)	1.6	27	22	4.3
σ_AEF_ (10^6^)	0.19	4.2	2.7	0.54
99% CI μ_AEF_ (10^6^)	1.6 < μ_AEF_ < 1.7	26 < μ_AEF_ < 28	21 < μ_AEF_ < 28	4.2 < μ_AEF_ < 4.3

It is interesting to note that the calculated AEF varies for different molecular vibrations. These differences in Raman scattering originate from the molecular orientation of BT over the Au surface, thereby enhancing certain modes more than others.^53^ This work focuses on the vibrational mode excited at 1077 cm^–1^. The vibrational mode located at 1077 cm^–1^ is chosen because of the combined in-plane bending of the benzene ring (δC–C–C) and the stretching mode of the C–S bond (ν C–S), thus being characteristic of a BT molecule covalently bonded through a thiol bond.^54,55^ The calculated AEF for this peak shows a strikingly low variance of roughly 4% over the recorded spectra with a mean AEF being 2.2 ± 0.1 × 10^7^ (99% confidence interval for *N* = 400). The minimal measured variance can be attributed to some aspects of the Au-NP@SiNC structure geometry and the actual measurement error. Therefore, the influence of the Au-NP diameter on the EF is evaluated in a numerical simulation and shown in Figure S14. Taking into account that the observed Au-NP diameter dispersity is 67 ± 6 nm, the calculated values for the EF show little difference between 50 to 80 nm diameter, as shown in Figure S14. The analytical enhancement factor (AEF), as extracted by experiment, and the enhancement factor (EF), calculated by numerical simulations, are different in a sense that the experimental result compares the value extracted from a surface to a value measured in the bulk liquid. This also requires the calculation of the *N*_Raman_/*N*_SERS_ ratio, as shown in (eq 2). If we compensate our calculations by dividing the AEF by the 5.8 × 10^4^ effect of *N*_RAMAN_/*N*_SERS_ (eq 2), the AEF found using Figure 8 is ∼10^3^–10^4^, which coincides with the simulated EF.

Measurements recorded on the non-annealed QD sample show the largest AEF values at 1025 and 1077 cm^–1^. This observation is also strengthened by the incident beam laser power study presented in Figure S15. Even at a power of 5 μW, which translates into an intensity of ∼3 W/cm^2^ and a surface energy density of ∼10 J/cm^2^, the most prominent peaks, those that are shown in Table 1, are still observed in the normalized, baseline-corrected, and smoothened spectra. Evidently, for the laser power studies, the signal-to-baseline (SNB) ratio, comparable to the peak-to-peak value, drops for lower laser powers. The number of detectable peaks decreases due to a relatively larger background. Arguably, the SNB drops significantly below a laser power of 50 μW or ∼ 100 J/cm^2^. Having a rather large and homogeneous AEF across the surface facilitates an improved SNB, yielding the largest number of detected peaks for the sample QD, visualized in Figure 7 by the downward pointing triangles. The detected peaks and the corresponding Raman modes are listed in Table S1.^54,55^

Table 2 summarizes a literature study of some novel SERS substrates that were fabricated in the past 2–3 years. The findings are characterized on the basis of their EF and the variance calculated in each work. When compared to other lithographically fabricated SERS substrates, we report a highly homogeneous SERS substrate with an exceptionally low variance (roughly 4%) and a relatively high EF (in the order of 10^7^).

**Table 2:** Comparison of Published SERS Substrates from a Lithographically Derived Fabrication Process^a^

S. No.	structure	EF	variance (%)	ref.
1	dendrimers	10^5^	8.4–9.4	(56)
2	nanoparticle branch	10^5^	5.4	(57)
3	nanowires	10^6^	13–15	(20)
4	Ag-NPs arranged on polymer pillars	10^8^	7.6	(58)
5	complex patterns	10^5^	6.0	(59)
6	silicon nanorods decorated with Au-NPs	10^5^	4.0–6.1	(60)
7	Au-coated silicon nanopillars	10^5^	5.2–6.9	(17)
8	Ag bowties	10^7^	12–13	(61)
9	dimer nanopillars	10^9^	3.2–5.6	(17)
10	nanochannel with self-assembled Au-NP	10^3^	13–41	(62)
11	Au nanocorals	10^6^	6.0	(63)
12	pillars replicated from diatom	10^7^	< 20	(64)
13	nanohoodoos	10^8^	6	(65)
14	this work	10^7^	∼4.0	

a The AEF and variance are stated accordingly.

Another interesting feature is the relative difference in peak values recorded at 0 cm^–1^. For the different samples as observed in Figure 7) and Figures S11 and S12, the relative change in position of the 0 cm^–1^ peak of the normalized spectra is evident. Initially, we hypothesized that this could primarily be because of a residual part of the scattered laser light passing through the notch filter. Typically when a high numerical aperture (NA; such as 0.9 in our case) is used, even the slightest deviation in the sample tilt may lead to this leak when the reflected light is of a highly specular nature. It is interesting to see that compared to the Au-film sample, for which a relatively large peak value at 0 cm^–1^ is recorded, no peak value is observed at 0 cm^–1^ when a QD sample is present in the beam area. Considering the above, we therefore speculate that the polycrystalline nature of Au and the optoelectronic 3d interband transition at ∼2.3 eV (539 nm) are responsible for the detection of the photons at a 0 cm^–1^ Raman shift. Light reflected from the sputtered Au surface can be predominantly attributed to geometrical and/or Rayleigh scattering.^66^ Solely considering elastic scattering, the QD samples may be of a more diffuse scattering nature due to the high surface roughness. The SiNC structure is ∼550 nm high with a conical opening angle of ∼14°. This is very different from the sputtered gold film, with a much smaller roughness (in the order of a few nm) due to the polycrystalline nature. Lastly, one also needs to consider the Au-NP on top of the SiNC, which might increase the diffuse nature of the elastically scattered light. Considering Figure S12, in 400 different positions, no elastically scattered light reaches the detector for the QD sample. However, in the case of the QD1000 sample, the 0 cm^–1^ peak is detected in all cases. We therefore argue that the observation is insensitive to the measurement location. Besides a high sensitivity for change in incidence angle of specular light, a high NA objective also collects a relatively large amount of the diffuse Rayleigh scattered light. The difference between the QD and QD_1000_ samples is that there is no c-Si present inside the SiNC structure of QD_1000_. Its structure is completely oxidized as shown in Figure 4c. Additionally, as silicon is an indirect bandgap material, one would expect phonon-assisted transitions to appear, yielding Raman-scattered photons. For the Si reference sample, SiNC reference sample, and QD samples, this is indeed the case. Large relative values were recorded around 520 cm^–1^, as observed in Figure 7.^67^ One could argue that, where the Si reference surface is a flat, smooth (viz. unstructured) surface, thus showing a combination of plain geometrical scattering and Raman scattering, the structured SiNC surfaces have a gradient diameter along the conical section that covers both the small scatterer (Rayleigh) limit but also could facilitate Mie scattering with a special emphasis on the anapole mode, as priorly discussed in the simulation section.^68,69^

## 4 Conclusions

A fabrication scheme was presented that deposits gold onto shell-insulated silicon nanocones. It is possible to deposit thin films of gold on selected faces of the nanocone structure for single and double depositions using discrete rotation glancing angle deposition. Furthermore, utilizing a quadruple deposition with discrete 90° substrate rotations between depositions and a substrate tilt angle of 85° facilitates the formation of spherical gold nanoparticles at the apices. A combination of reflectance spectra measurements and finite difference time domain simulations shed light on the optical modes that can be addressed to deliver a surface enhanced Raman effect. A substrate was fabricated that showed a high enhancement factor being (2.2 ± 0.1) × 10^7^ (99% confidence interval for *N* = 400) with a remarkably low 4% EF variance over the entire substrate useful for SERS applications.

## Supporting Information Available

Additional SEM double deposition anneal; annealed and etched in HF; histogram Au-NP diameter; automated measurement; annealing different temperatures; annealing in air and vacuum; quadruple deposition 20 nm; scattering and absorption cross section; reflectance spectra substrate thickness; Raman spectra different samples; raw spectra; additional surface maps; Au-NP diameter vs enhancement; laser power Raman study; Raman band table (PDF)

## ABBREVIATIONS

SERS: surface-enhanced Raman spectroscopy
EF: enhancement factor
FDTD: Finite-Difference Time-Domain
DTL: displacement Talbot lithography
GLAD: glancing angle deposition
Au-NPs: gold nanoparticles
SiNC: silicon nanocones
Au-NP@SiNC: gold nanoparticles on the apices of silicon nanocones
AEF: analytical enhancement factor
RIE: reactive ion etching
N_2_: nitrogen
BT: benzenethiol
t-SiO_2_: thermally grown silicon dioxide
c-Si: crystalline silicon
DR-GLAD: discrete rotation glancing angle deposition
QD: silicon nanocone with gold nanoparticle
QD_800_: silicon nanocone with gold nanoparticles annealed in air at 800 °C for 12 h
QD_1000_: silicon nanocone with gold nanoparticles annealed in air at 800 °C for 12 h
FIB: focused ion beam etching
EDX: energy-dispersive X-ray spectroscopy
SEM: scanning electron microscopy
NA: numerical aperture
