# Supplementary material for: Low-Variance Surface-Enhanced Raman Spectroscopy Using Confined Gold Nanoparticles over Silicon Nanocones
Source: ACS Appl Nano Mater. 2023 May 20;6(11):9657–69. doi: 10.1021/acsanm.3c01249 (PMC10262153; doi:10.1021/acsanm.3c01249)
Supplement: Supplementary file 1 — an3c01249_si_001.pdf [file an3c01249_si_001.pdf]

Supporting Information: Low-variance SERS Using Confined Gold Nanoparticles over Silicon Nanocones

*Dirk Jonker *‡[a], Ketki Srivastava *‡[b], Marta Lafuente [a], Arturo Susarrey-Arce [a], Ward van der Stam [c], Albert van den Berg [b], Mathieu Odijk [b], Han J.G.E Gardeniers [a]*

[a] Mesoscale Chemical Systems, MESA+ Institute, University of Twente, P.O. Box 217, 7500 AE Enschede, The Netherlands.

[b]BIOS, MESA+ Institute, University of Twente, P.O. Box 217, 7500 AE, Enschede, The Netherlands

[c] Inorganic Chemistry and Catalysis, Institute for Sustainable and Circular Chemistry and Debye Institute for Nanomaterial Science, Utrecht University, Universiteitsweg 99, 3584 CG Utrecht, The Netherlands

‡These authors contributed equally

*Corresponding Author: d.jonker@utwente.nl; k.srivastava@utwente.nl

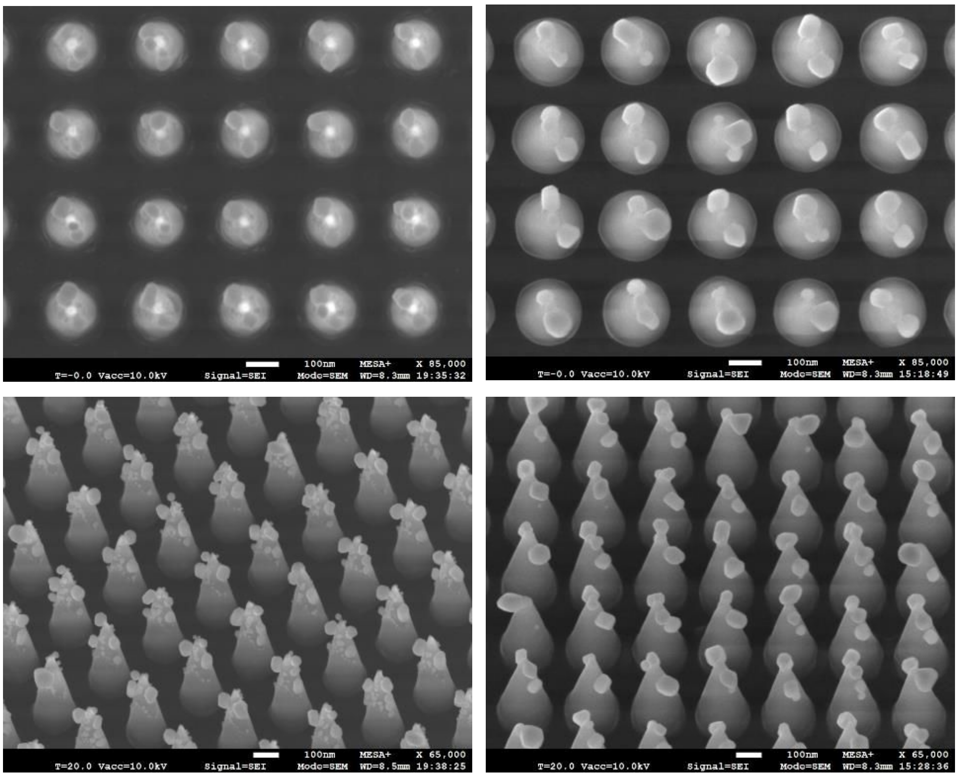

Figure S1. Additional SEM micrographs of SiNCs containing Au-NPs. A double deposition of 10 nm Au was performed at $\theta = 75°$ and $d\varphi = 180°$. The left column contains SEM images after annealing in air at 800 ˚C for 10 minutes, whereas the right column contains SEM images collected after annealing in air at 1000 ˚C for 1 hour. The scale bars represent 100 nm.

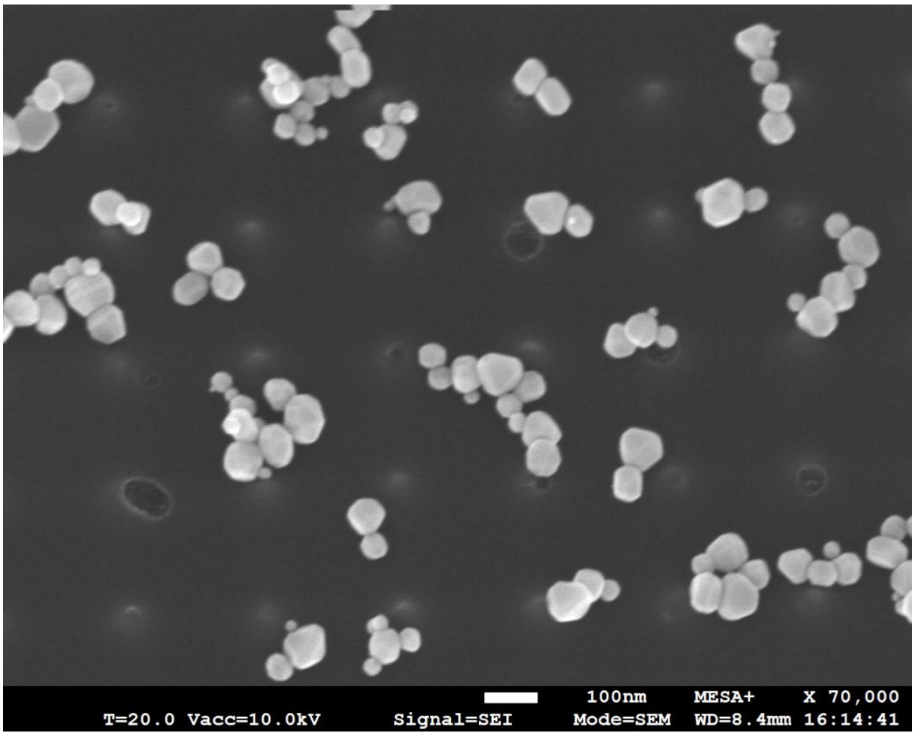

Figure S2. SEM micrograph of the SiNCs array that was annealed at 1000 ˚C for 1 hour and dipped in an aqueous 50% HF solution for 5 minutes. Some small features are left at the locations which used to contain SiNC structures together with Au-NPs. The Au-NPs show facets characteristic for Au-NP that have been annealed and cooled down relatively slow. The scale bars represent 100 nm.

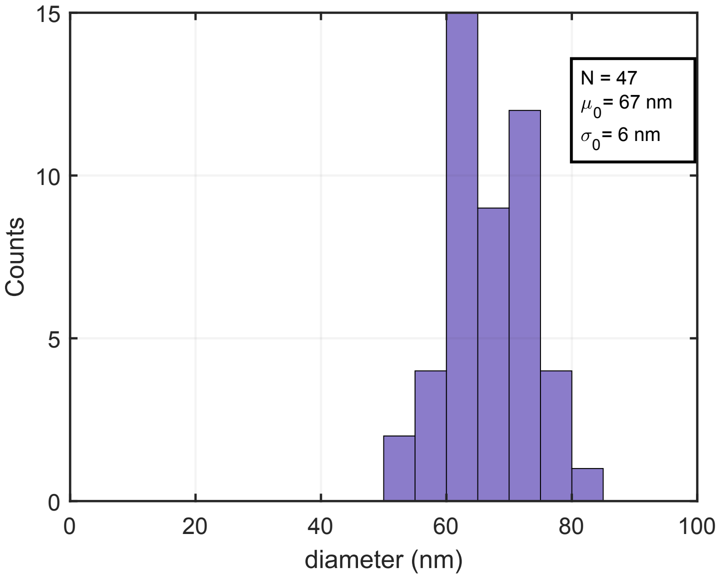

Figure S3. Histogram plot of measured Au-NP diameters for the quadruple deposition of 10 nm Au under θ= 85˚.

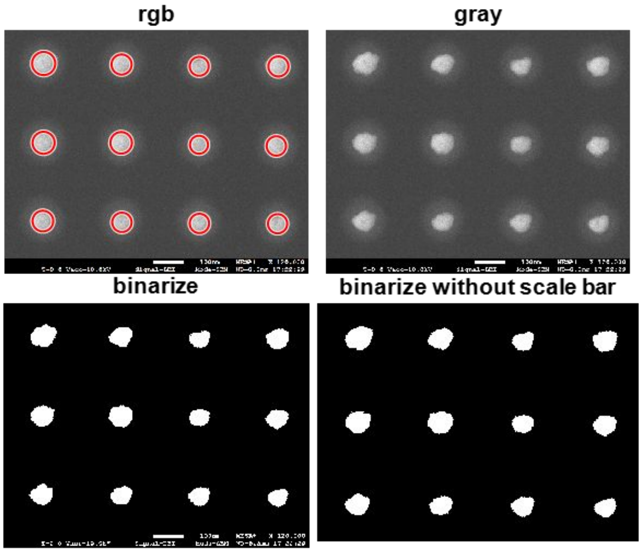

Figure S4. Example of top-view SEM images for automated measurement of Au-NP diameters.

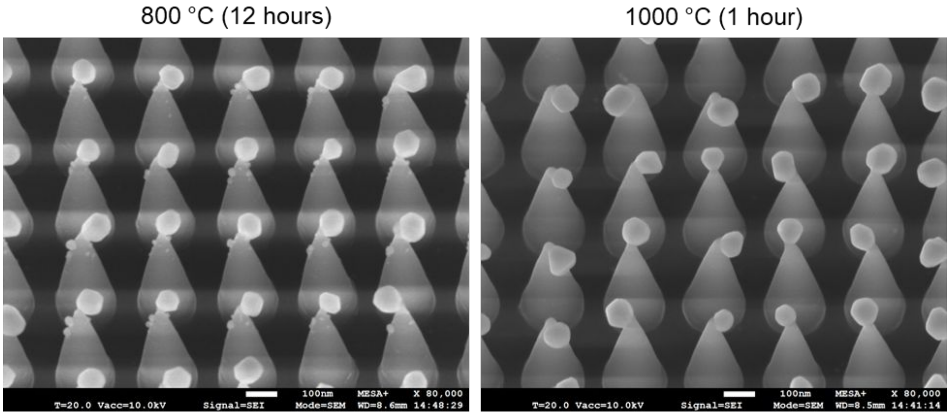

Figure S5. SEM images collected for Au-NPs on SiNC fabricated through quadruple deposition of 10 nm Au at $\theta = 85°$ through $d\varphi = 90\,°$ rotations. SEM images were recorded for two different annealing procedures: an annealing step in air at 800 ˚C for 12 hours or an annealing step in air at 1000 ˚C for 1 hour. The scale bars represent 100 nm.

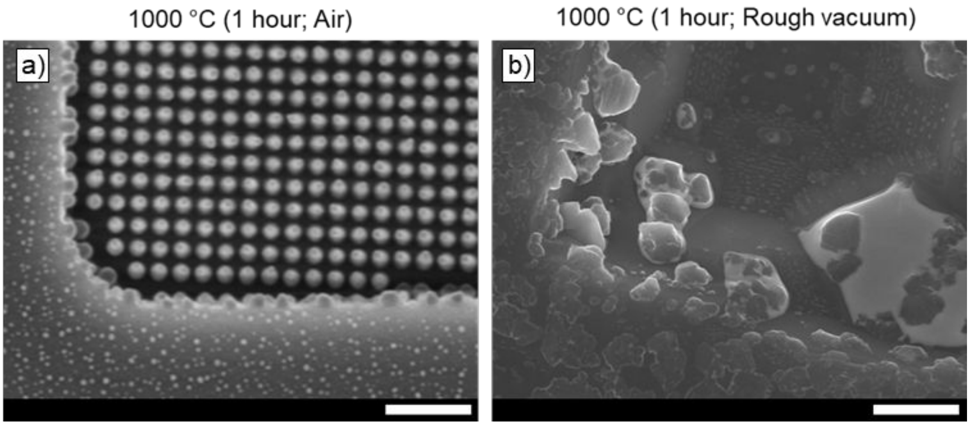

Figure S6. SEM images for two different annealing procedures a) annealing step in air at 1000 ˚C for 1 hour and b) annealing step in rough vacuum conditions under a nitrogen flow at 1000 ˚C for 1 hour. The scale bars represent 1000 nm.

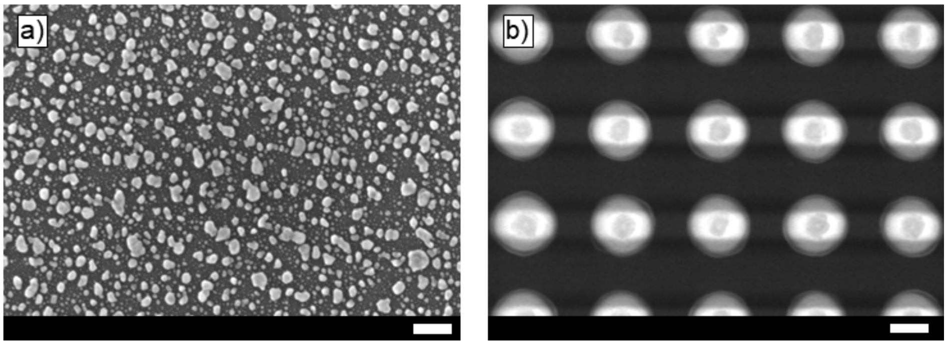

Figure S7. SiNC fabricated through quadruple deposition of 10 nm Au at $\theta = 85°$ through $d\varphi = 90$ ° rotations over the a) flat, and b) SiNC surface. The scale bar represents 100 nm.

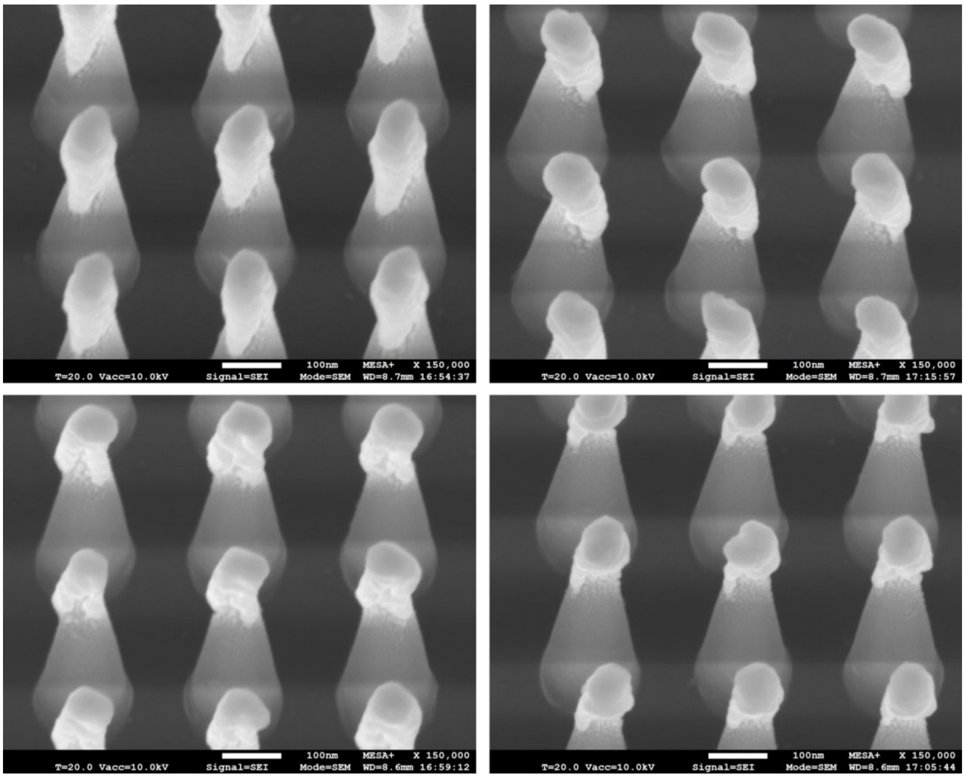

Figure S8. SEM images collected for Au-NPs on SiNC fabricated through quadruple deposition of 20 nm Au at $\theta = 80°$ through $d\varphi = 90\ °$ rotations. SEM i-mages were collected for 4 discrete rotations, visualizing the DR-GLAD process on different faces of the SiNC structure. The scale bars represent 100 nm.

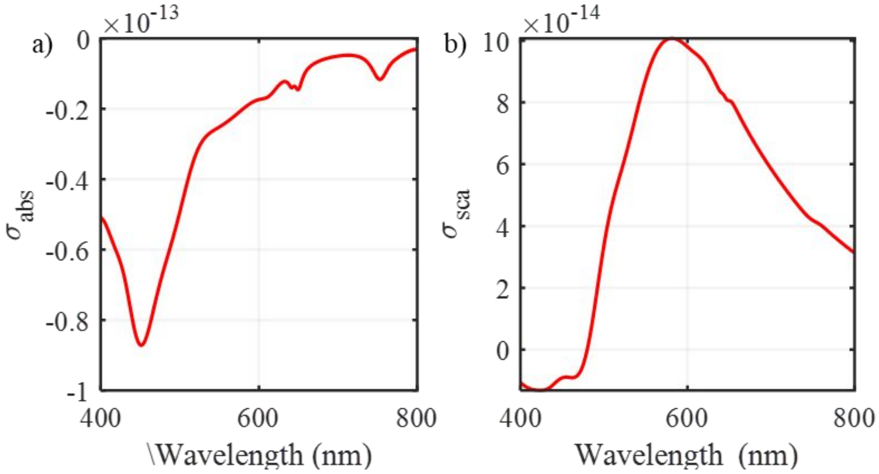

Figure S9. a) scattering and b) absorption cross-sections of the QD sample conSdering a single particle using a perfectly matched layer boundary condition on all exterior boundaries.

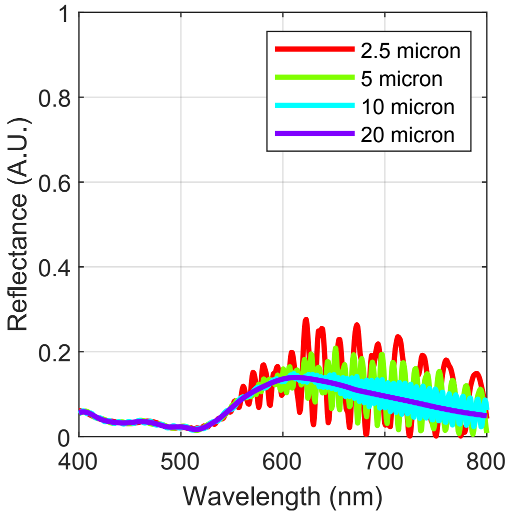

Figure S10. Reflectance spectra of QD sample for different bulk silicon domain heights. Fabry-Perrot modes are evidenced by periodic modulations of the recorded graphs at longer wavelengths. Again, these modulations vanish and move to larger wavelengths with increasing bulk c-Si domain size.

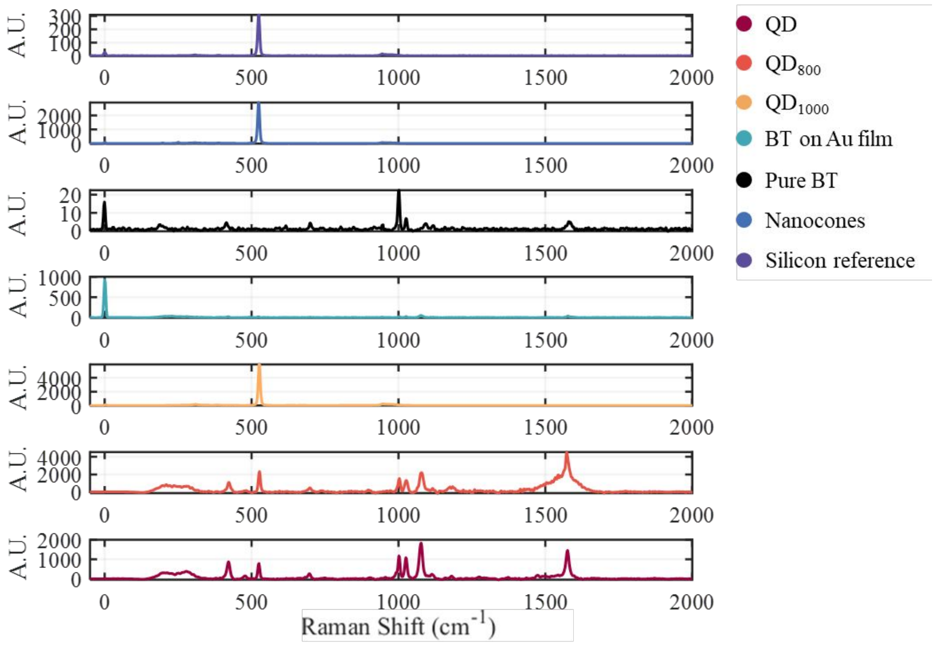

Figure S11. Smoothed and baseline corrected Raman spectra recorded for the different sample treatments. Every presented spectrum is the average of 10 recorded spectra. Note that the y-axis scale (A.U.) is different for sample treatments to support graphical clarity.

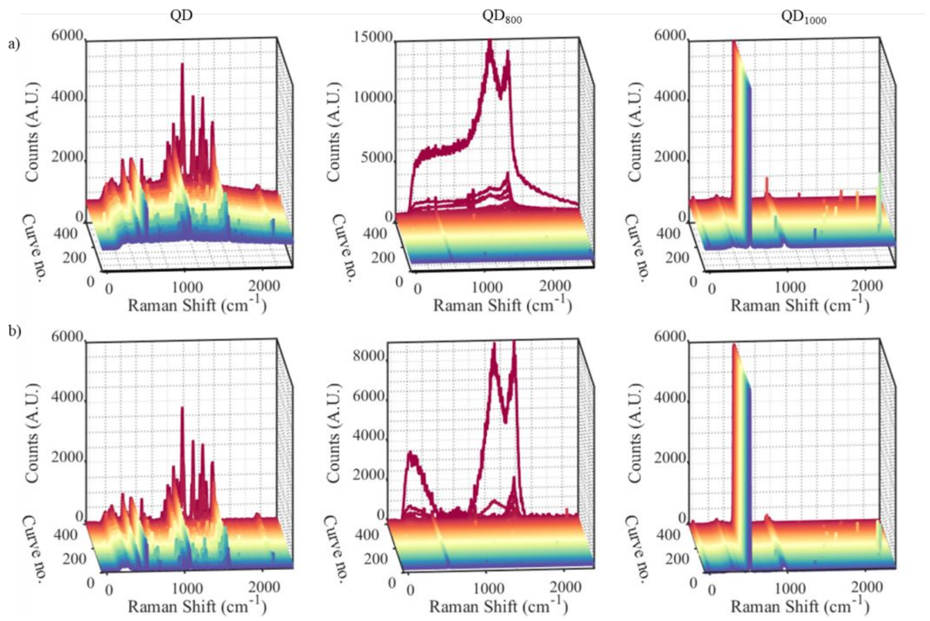

Figure S12. a) Raw, and b) baseline corrected, and smoothed Raman spectra, from which the normalized spectra in Figure 9.8 and the surface maps in Figure 9.9 are derived for the QD, QD_{800}, and QD_{1000} samples. A single measurement was taken for every spectrum.

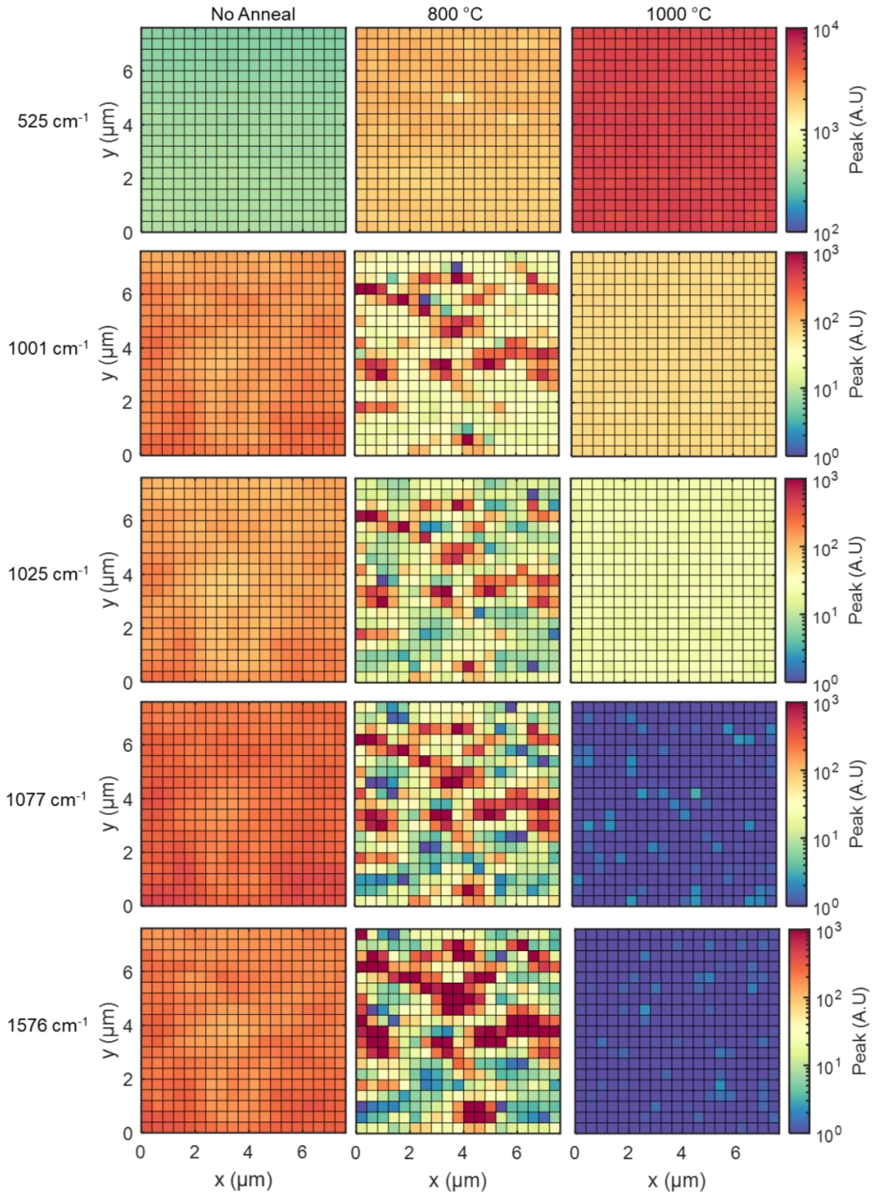

Figure S13. Additional surface maps displaying the peak values of baseline corrected and smoothed spectra at 5 distinct Raman bands being the 525, 1001, 1025, 1077, and 1576 cm^{-1} bands for the No anneal (QD), 800 ˚C (QD_{800}), and 1000 ˚C (QD_{1000}) sample recorded over an 8×8 μm^2 surface using 20×20 grid points. A single measurement was taken for every pixel.

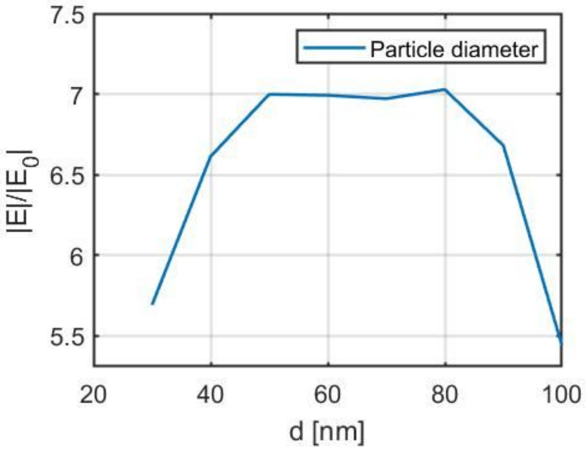

Figure S14. Maximum normalized electric field magnitude plotted for varying AuNP diameter, d, on SiNC structures, mimicking the situation as in the QD sample simulated at an excitation wavelength of 633 nm. The calculated values show little variance between 50 to 80 nm diameter.

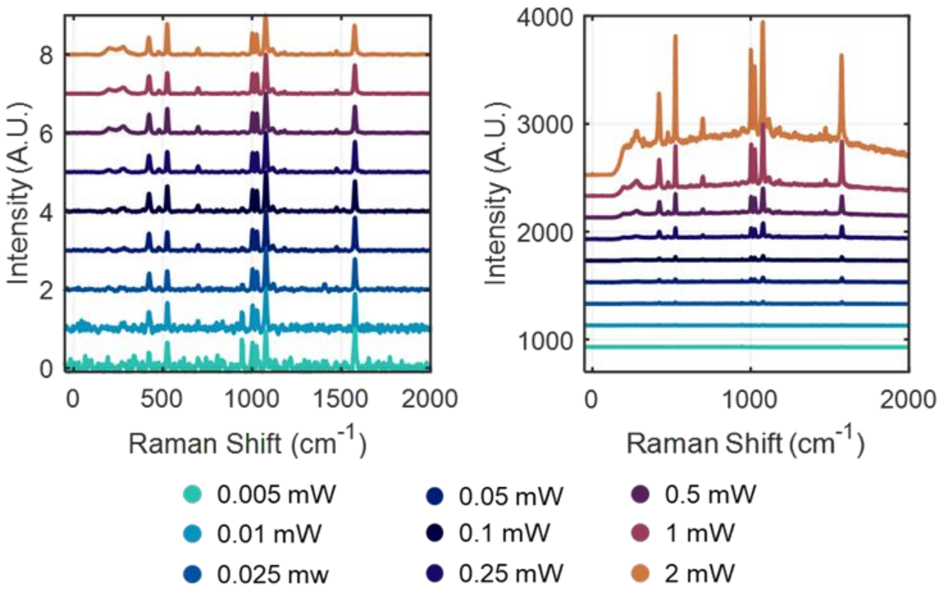

Figure S15. Normalized, baseline corrected, and smoothened Raman spectra, together with the raw recorded signals at different excitation laser powers. The spectra were collected with a 3s integration time. Every presented spectrum is the average of 10 recorded spectra. An offset is given for clarity.

Table S1 Detected Raman bands for the measured samples. Str= stretch, bnd=bend, i.p.= in-plane, o.o.p.= out of plane.

Raman shift (cm^{-1})							Mode
Si ref.	Pure BT	SiNC ref.	BT on Au Film	QD_{1000}	QD_{800}	QD	
	193				210	202	
			230				
						276	Au-S str
	415				423	422	i.p. C-S str
					482	478	o.o.p. C-C-C bnd
525		523		527	527	525	LTO (c-Si)
	617						
	700				698	696	i.p. C-C-C ring bnd
	918				897	903	o.o.p. C-C-H bnd
943							2TO (c-Si)
	1002				1003	1001	i.p. C-C-C ring bnd
	1025				1027	1025	i.p. C-H ring bnd
	1092		1075		1079	1077	C-C-C bend +C-S str
	1119					1113	i.p. C-C-H bend + C-C-S bnd
	1158				1184	1180	i.p. C-H ring bnd
						1275	i.p. C-H ring bnd
						1373	i.p. C-H ring bnd
						1474	i.p. C−C str + C−H bnd + C−S str
	1582		1577		1572	1576	symm i.p. C−C ring str

Effect of arPLS Baseline Correction on the Observed Variance in the Spectroscopy Study

Baseline correction was applied using the arPLS algorithm to each individual spectrum in this study, where λ and p values were selected with great caution. The resulting behavior of the baseline is dependent on these parameters. Fig. S16 shows the outcomes of various λ values. It can be deduced that arPLS baseline correction adds an additional variance term, as it involves subtracting the baseline and creating an offset. The offset value is contingent on the baseline of each individual spectrum, which is inherently stochastic. Consequently, a random offset is introduced for each spectrum, leading to an additional variance term. As the total variance is a linear combination of all individual variance components, the use of arPLS algorithm can only increase variance and not reduce it.

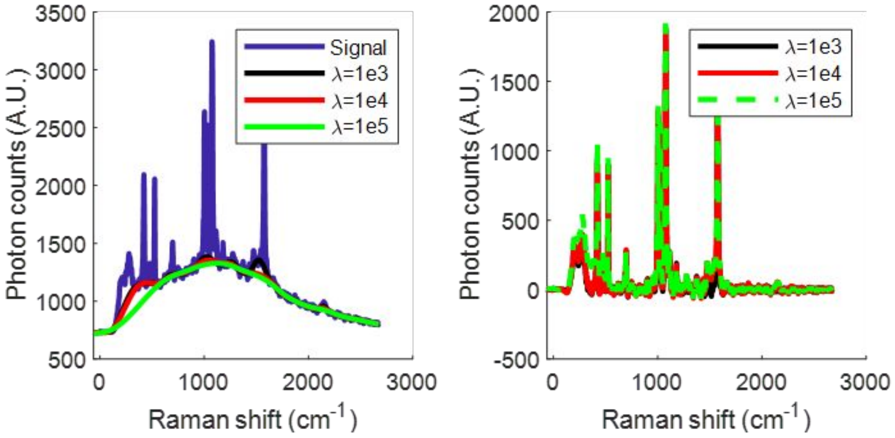

Figure S16. Raw signal, and baseline correction with different λ values in the left panel, and baseline corrected spectra in the right panel.

Effect of the Moving Average Filter on the Observed Variance in the Spectroscopy Study

The smoothening considers a moving mean (box-car average) with window size N. This means that:

$$y_k = \frac{1}{N}\sum_{i=k-N+2}^{k+N-2} x_i$$

Where y_k is the newly (filtered value), x_i is the recorded data point, and N is the window size. With N=3, only the nearest neighbour (k-1, k, k+1) data points are considered. Similar to the argument made for the baseline correction, the smoothening step is also applied to each individual spectrum. Therefore, the offset created in a single data point y_k is also stochastic by nature, although a filter is applied. To evidence this we calculated the AEF maps for different values of N, the result is displayed in Fig. S17. It is evident from the results that an increase in N yields an increase in σ. Although this observation may seem counterintuitive, we suggest that a larger N entails a higher number of data points and, consequently, a larger individual offset for each curve. Nevertheless, as total variance represents a linear combination of individual variance components, it results in an overall increase in the observed variance.

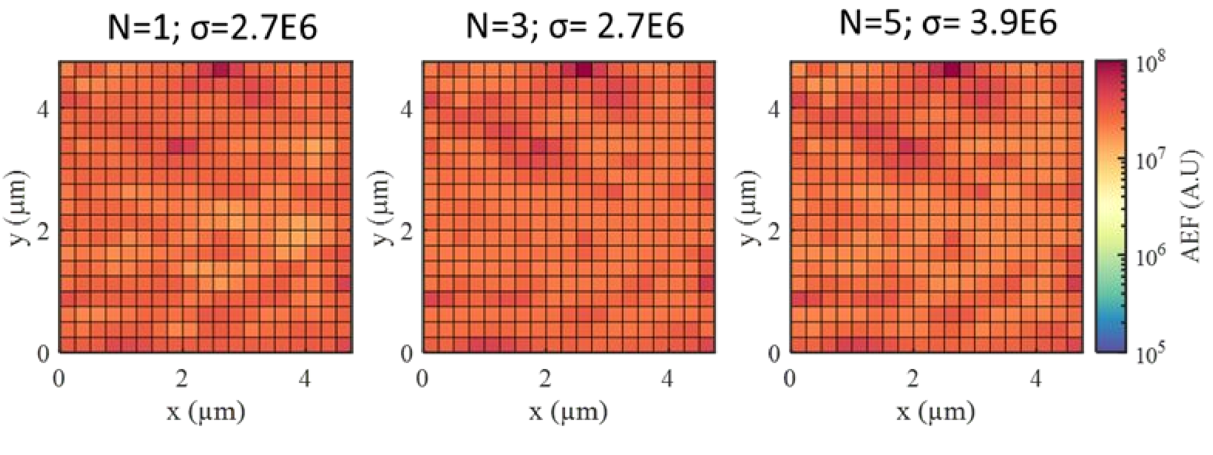

Figure S17. The calculated AEF for the 1077 cm^{-1} peak on the QD sample where different moving average window size were applied. The window size, N, and calculated standard deviation, σ, are stated in the panel headers.
